# Physiological analysis of the effect of altitudinal gradients on *Leymus secalinus* on the Qinghai-Tibetan Plateau

**DOI:** 10.1371/journal.pone.0202881

**Published:** 2018-09-05

**Authors:** Guowen Cui, Bing Li, Wenhua He, Xiujie Yin, Shengyong Liu, Lu Lian, Yaling Zhang, Wenxue Liang, Pan Zhang

**Affiliations:** 1 Department of Grassland Science, College of Animal Science and Technology, Northeast Agricultural University, Harbin, China; 2 Grassland Supervision Station, Qiqihar, China; 3 Chinese Grassland Society, Beijing, China; University of Innsbruck, AUSTRIA

## Abstract

On the Qinghai-Tibetan Plateau, the high-altitudinal gradients can negatively affect plant distribution and limit plant growth and reproduction. *Leymus secalinus* (Georgi) Tzvel. is an important forage crop on the Qinghai-Tibetan Plateau and has an excellent ability to fix sand and improve soil. To evaluate the effect of altitude on the physiological characteristics of *L*. *secalinus* on the Qinghai-Tibetan Plateau, we measured the lipid peroxidation; chlorophyll *a* (Chl *a*), chlorophyll *b* (Chl *b*), total carotenoid (Car), soluble protein, proline and soluble sugar contents; and the activities of superoxide dismutase (SOD), catalase (CAT) and peroxidase (POD) in leaves from eight different altitudes in Minhe County and Huangzhong County. The leaves were collected at the initial bloom stage, and the average vertical distance between two adjacent collection sites was approximately 100 meters. The reduction in Chl *a* and Chl *b* contents and the increase in Car contents can allow plants to weaken their light absorption and avoid photodamage to the chloroplast. The decrease in malondialdehyde (MDA) content associated with lower lipid peroxidation, and the changes of CAT, SOD and POD activities reflect a higher reactive oxygen species (ROS) scavenging capacity in high-altitude plants. The increase in proline and soluble sugar contents with elevation suggests that proline and soluble sugar may play a key role in the osmotic adjustment of plants in alpine regions. The results suggested that altitudinal gradients negatively affect *L*. *secalinus* on the Qinghai-Tibetan Plateau and that the adaptation mechanism and survival strategies of *L*. *secalinus* were attributed to the combined effects of multiple protective strategies.

## Introduction

In natural environments, plants must overcome many abiotic stresses, such as drought, salt, cold, ultraviolet radiation (UV) and altitude, to complete their life cycles [1]. Altitudinal gradients cause the climate and environment to differ greatly within a short vertical distance by decreasing air temperature, total atmospheric pressure and partial pressure of all atmospheric gasses and by increasing radiation in the forms of incoming solar radiation, outgoing nighttime thermal radiation, and UV radiation [2, 3]. Plants growing along high-altitudinal gradients experience interacting stresses, including weathering, dehydration and low temperature [4]. Due to the influence of altitude, the growing period of plants is shortened by low temperatures at high altitudes, and light limitation at high altitudes reduces plant growth and reproduction compared to the light conditions that occur at low altitudes [5].

The Qinghai-Tibetan Plateau, the highest plateau in the world, plays an important role in climate change in Asia and even the whole world [6]. However, the grassland ecosystem on the Qinghai-Tibetan Plateau has become increasingly degraded during recent years with the increase in human activity, overgrazing and climate change [7]. The environment of the Qinghai-Tibetan Plateau is adverse for plant growth. The high-altitudinal gradient can limit plant growth and reproduction via strong solar UV-B radiation, resulting in a reduction in the photosynthetic rate by bleaching chlorophyll *a* (Chl *a*) and damaging the photosynthetic apparatus [8]. With increasing altitude, the mean fractions of total biomass allocated to aboveground plant parts decreased to promote carbon gain and nutrient uptake [9]. In addition, the dramatic change in adaptability within the plant that developed gradually over a long period of time to allow survival in adverse environments not only is manifested in the morphological structure of the plant but also includes the antioxidant defenses, hormone levels, osmotic adjustment, and increased saturation level of cell membrane lipids in the plant; such adaptation is the result of a long-term evolutionary process in concert with stress conditions [10, 11].

*Leymus secalinus* (Georgi) Tzvel. is a perennial rhizomatous grass that has strong resistance to saline-alkaline soils, drought, cold, and trampling and is distributed widely in China and in Mongolia and Japan [12]. This grass occurs in grasslands, sandy grasslands, mountain slopes, farmlands and roadsides and is a typical clonal plant (the branching type of belowground rhizomes is sympodial) in moving sand dunes that is known to have an extraordinary ability to fix sand dunes [13]. *L*. *secalinus* is utilized not only as an excellent forage crop but also for soil improvement and as genetic material to improve crop resistance by hybridization with wheat or other cereal crops [14].

In China, grassland degradation in arid and semiarid areas has become a serious environmental problem due to climate variability and human disturbance [15]. If no effective measures are taken to solve the forage shortage that has accompanied the increase in herbivorous livestock on the Qinghai-Tibetan Plateau, then grazing, a fundamental practice used by local people to satisfy their daily needs for a very long period of time, will exacerbate the degradation. High-yielding and high-quality herbs, such as *L*. *secalinus*, adapted to the harsh conditions in this region are urgently needed to balance the relationship between ecological environmental protection and livestock development. However, little is known about the physiological adaptability of *L*. *secalinus* under the complex environmental stresses on the Qinghai-Tibetan Plateau. The aim of this study was to explore the possible changes in physiology in *L*. *secalinus* along different altitudinal gradients on the Qinghai-Tibetan Plateau by measuring the contents of MDA, Chl *a*, Chl *b*, Car, soluble protein, proline and soluble sugar, and the activities of SOD, CAT and POD.. The results may reveal the adaptation mechanism and survival strategies of *L*. *secalinus* under complex environmental stresses in alpine regions.

## Material and methods

### Ethics statement

This research was approved by the Grassland Station of Minhe County and Huangzhong County in China.

### Experimental design and sample collection

The experiment was conducted in Minhe County (MH, 35°45′~36°26′ N, 102°26′~103°04′ E) and Huangzhong County (HZ, 36°13′~37°03′ N, 101°09′~101°54′ E). Although these two counties are both located in the northeast of the Qinghai-Tibet Plateau in Qinghai Province of China, MH has a plateau continental arid climate with an annual average temperature of 9.4°C, average annual precipitation of 362.8 mm and annual accumulated sunlight of 2404.8 h, while the HZ has an alpine semiarid climate with an annual average temperature of 6.2°C, average annual precipitation of 398.9 mm and annual accumulated sunlight of 2617.3 h. The soil in the eight experimental sites is chestnut soil. The soil properties including pH, organic matter, available nitrogen, available phosphorus, and available potassium are 8.02, 16.80 g kg^-1^, 90.67 mg kg^-1^, 22.50 mg kg^-1^, and 165.76 mg kg^-1^ at MH and 7.90, 18.10 g kg^-1^, 103.64 mg kg^-1^, 20.80 mg kg^-1^, and 170.72 mg kg^-1^ at HZ, respectively. To evaluate the effect of altitudinal gradients on *L*. *secalinus*, four populations with the same natural habitat in MH or HZ were chosen as individual experiment sites (Table 1). The average vertical distance between two adjacent experimental sites was approximately 100 meters, and all the sites were located in natural grassland without grazing or any other human interference.

**Table 1:** Location and altitude of the experimental sites.

Site	Altitude (m)	Latitude (N)	Longitude (E)	Location
MH1	1872	36°18′	102°45′	Minhe County
MH2	1978	36°16′	102°44′	Minhe County
MH3	2080	36°14′	102°41′	Minhe County
MH4	2185	36°14′	102°39′	Minhe County
HZ1	2613	36°22′	101°45′	Huangzhong County
HZ2	2718	36°24′	101°43′	Huangzhong County
HZ3	2826	36°28′	101°50′	Huangzhong County
HZ4	2935	36°21′	101°44′	Huangzhong County

Samples were collected in July of every year from 2014 to 2016 with the five-point sampling method. Twenty-five expanded and intact flag leaves of a similar age in the initial bloom stage were harvested as one biological replicate, and five samples were collected at each site. Then, the leaves were wrapped in aluminum foil, immediately immersed in liquid nitrogen, and stored at -80°C for laboratory analysis. All the assays were repeated four times.

### Determination of lipid peroxidation

Lipid peroxidation was measured using the thiobarbituric acid (TBA) method described by Heath and Packer, with some modifications [16]. The leaves were ground in 8 ml of 10% (w/v) trichloroacetic acid (TCA) with a mortar. The mixture was centrifuged at 4,000 × g for 10 min at 4°C, and 2 ml of the supernatant was mixed with 2 ml of 6% TBA in 10% TCA. The mixture was heated at 100°C for 15 min and then cooled quickly to room temperature in an ice bath. The cooled mixture was centrifuged at 4,000 × g for 10 min. The absorbance of the supernatant was measured at 450, 532, and 600 nm. Malondialdehyde (MDA) content was calculated with the equation MDA (μmol L^-1^) = 6.45 (A532—A_600_) − 0.56A_450_ and expressed as nmol g^−1^ dry weight (DW; fresh leaves were dried at 80°C for 48 h).

### Determination of leaf photosynthetic pigments

To measure leaf pigments, the leaves were ground in a mortar with 10 ml 95% (v/v) ethanol and then filtered into a 25 ml volumetric flask. The slurry was re-extracted until the green color disappeared. The absorbance of chlorophyll *a* (Chl *a*), chlorophyll *b* (Chl *b*), and total carotenoids (Car) was measured at 665 nm, 649 nm, and 470 nm, respectively, and their concentrations (mg g^−1^ DW) were calculated according to Wellburn and Lichtenthaler [17] with the following equations:

$$\text{Chl}\;a=\left(13.95{\text{A}}_{665}-6.88{\text{A}}_{649}\right)\times \text{volume}\;\text{of}\;\text{supernatant}/\text{sample}\;\text{weight}/1000$$

$$\text{Chl}\;b=\left(24.96{\text{A}}_{649}-7.32{\text{A}}_{665}\right)\times \text{volume}\;\text{of}\;\text{supernatant}/\text{sample}\;\text{weight}/1000$$

$$\text{Car}=\left(1000{\text{A}}_{470}-2.05\text{Chl}\;a-114.8\text{Chl}\;b\right)/245\times \text{volume}\;\text{of}\;\text{supernatant}/\text{sample}\;\text{weight}/1000.$$

### Determination of soluble protein content and antioxidant enzyme activities

Approximately 0.2 g of each leaf sample was homogenized on ice with a mortar and pestle in 8 ml 0.1 M sodium phosphate buffer (PBS) (pH 7.8) containing 1% polyvinylpyrrolidone (PVP). The homogenate was centrifuged at 12,000 × g for 15 min at 4°C. The supernatant was diluted to 25 ml for the following protein and enzyme assays. The soluble protein content was determined according to Bradford’s method using bovine serum albumin as a standard [18].

Superoxide dismutase (SOD, EC 1.15.1.1) activity was measured by the nitroblue tetrazolium (NBT) method [19]. The reaction mixture (3 ml) contained 1.5 ml of 50 mM PBS (pH 7.8), 0.3 ml of 130 mM L-methionine, 0.3 ml of 750 μM NBT, 0.3 ml of 100 μM EDTA-Na_2_, 0.3 ml of 20 μM riboflavin and 0.3 ml of enzyme extract. The reaction was started by placing tubes below 4000 lx (light intensity) fluorescent lamps for 30 min, and the absorbance was measured at 560 nm. One unit of SOD activity was defined as the amount of enzyme that inhibited the rate of photoreduction of NBT by 50% and was expressed as U mg^-1^ protein.

The activity of catalase (CAT, EC 1.11.1.6) was measured following the method of Maehly [20], with some modifications [21]. The reaction mixture (3 ml) comprised 1.5 ml 50 mM PBS (pH 7.0), 0.3 ml 200 mM H_2_O_2_ and 0.2 ml enzyme extract. The reduction in absorbance of the reaction solution due to the decomposition of H_2_O_2_ was measured at 240 nm. One unit of CAT activity was defined as an absorbance decrease of 0.1 per minute.

The activity of peroxidase (POD, EC 1.11.1.7) was measured according to Zaharieva et al. [22], with minor modification. The POD reaction solution (5 ml) contained 50 mM PBS (pH 5.5), 2% H_2_O_2_, 50 mM guaiacol, and 0.1 ml enzyme extract. Changes in absorbance of the reaction solution at 470 nm were determined every 30 s. One unit of POD activity was defined as the amount of enzyme needed to decompose 1 mol of H_2_O_2_ per min at 25°C.

### Determination of free proline and soluble sugar content

Proline content was determined spectrophotometrically using the ninhydrin method described by Bates et al. [23], with some modifications. Leaves were homogenized with 8 ml of 3% aqueous sulfosalicylic acid and centrifuged at 3,000 × g for 10 min. The supernatant (2 ml) was mixed with 2 ml of acidic ninhydrin reagent and 2 ml of glacial acetic acid. The reaction mixture was heated in a water bath at 100°C for 1 h. The tubes were placed into ice immediately to stop the reaction, 4 ml of toluene was added, and the solution was mixed vigorously for 10~30 s. Absorbance of the toluene layer was read at 532 nm, and the proline content was calculated using a standard curve and expressed as μmol g^-1^ DW.

The soluble sugar content was determined following the method of Dreywood [24], with some modifications. Leaf samples (0.2 g) were extracted with 10 ml distilled water in a boiling water bath for 30 min and filtered into a volumetric flask. This extraction process was repeated twice. Filtrates (0.5 ml) were mixed with 1.5 ml distilled water, 5 ml of 98% sulfuric acid and 0.5 ml freshly prepared anthrone solution (1 g of anthrone dissolved in 50 ml of ethyl acetate). The reaction mixture was incubated in boiling water for 1 min. After the mixture cooled to room temperature, the absorbance at 630 nm was measured in a spectrophotometer, and the soluble sugar content was calculated according to a standard curve of glucose.

### Statistical analysis

All the assays described above were repeated at least four times on five biological replicates. The samples collected in each year were measured individually. Data are shown as the mean ± SD of three years. The data were subjected to analysis of variance (ANOVA) using IBM SPSS Statistics version 19.0 to test for significant differences, and the least significant differences (LSDs) of the means were determined at the level of significance (defined as α = 0.05). Curve fitting between the physiological parameters and altitudes was performed by means of nonlinear regression using Sigmaplot 10.0 software (Systat Software, Inc., California).

## Results

### Changes in lipid peroxidation along altitudinal gradients

**Fig 1:**
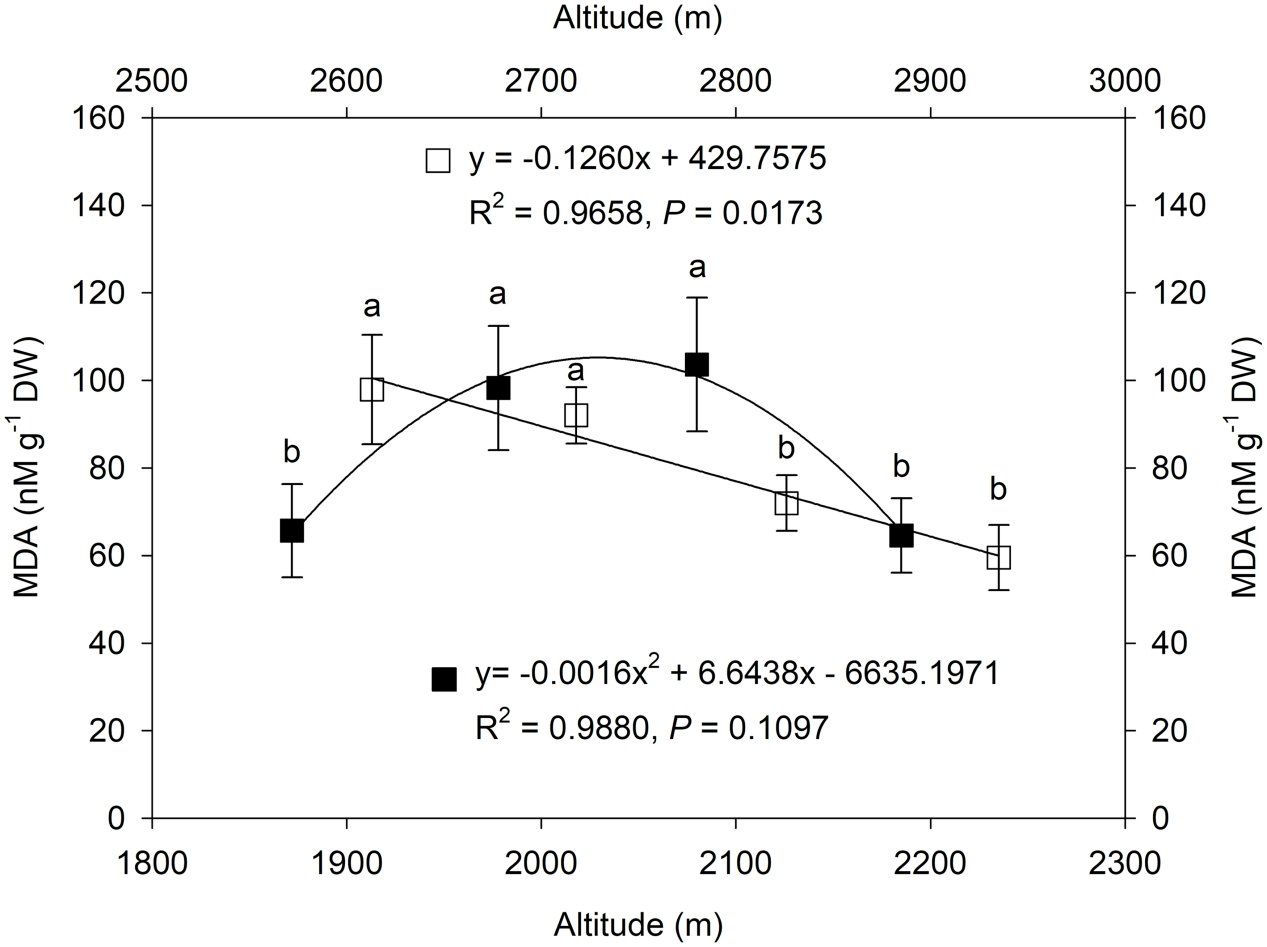
Changes in the MDA level along altitudinal gradients in Minhe (MH = ■) and Huangzhong County (HZ = □). The trendline, regression formula, coefficient of determination and P-value for sites in MH and HZ are shown. Values are shown as the mean ± SD of three years. Different letters indicate that the mean values are significantly different among the treatments (α = 0.05).

To investigate whether altitudinal gradients will change oxidative damage in *L*. *secalinus*, we examined the malondialdehyde (MDA) content in *L*. *secalinus* as an indicator of lipid peroxidation. Each of the four sites located in Minhe County (MH) or Huangzhong County (HZ) was examined, and the sites were then compared (Fig 1). The MDA content first increased and then decreased with an increase in altitude in MH, and MH3 had the highest MDA content, which was 1.6 times higher than that in both MH1 and MH4. In HZ, the levels of MDA showed a significant negative correlation with elevation, and the correlation coefficient was 0.9827.

### Changes in photosynthetic pigment content along altitudinal gradients

**Fig 2:**
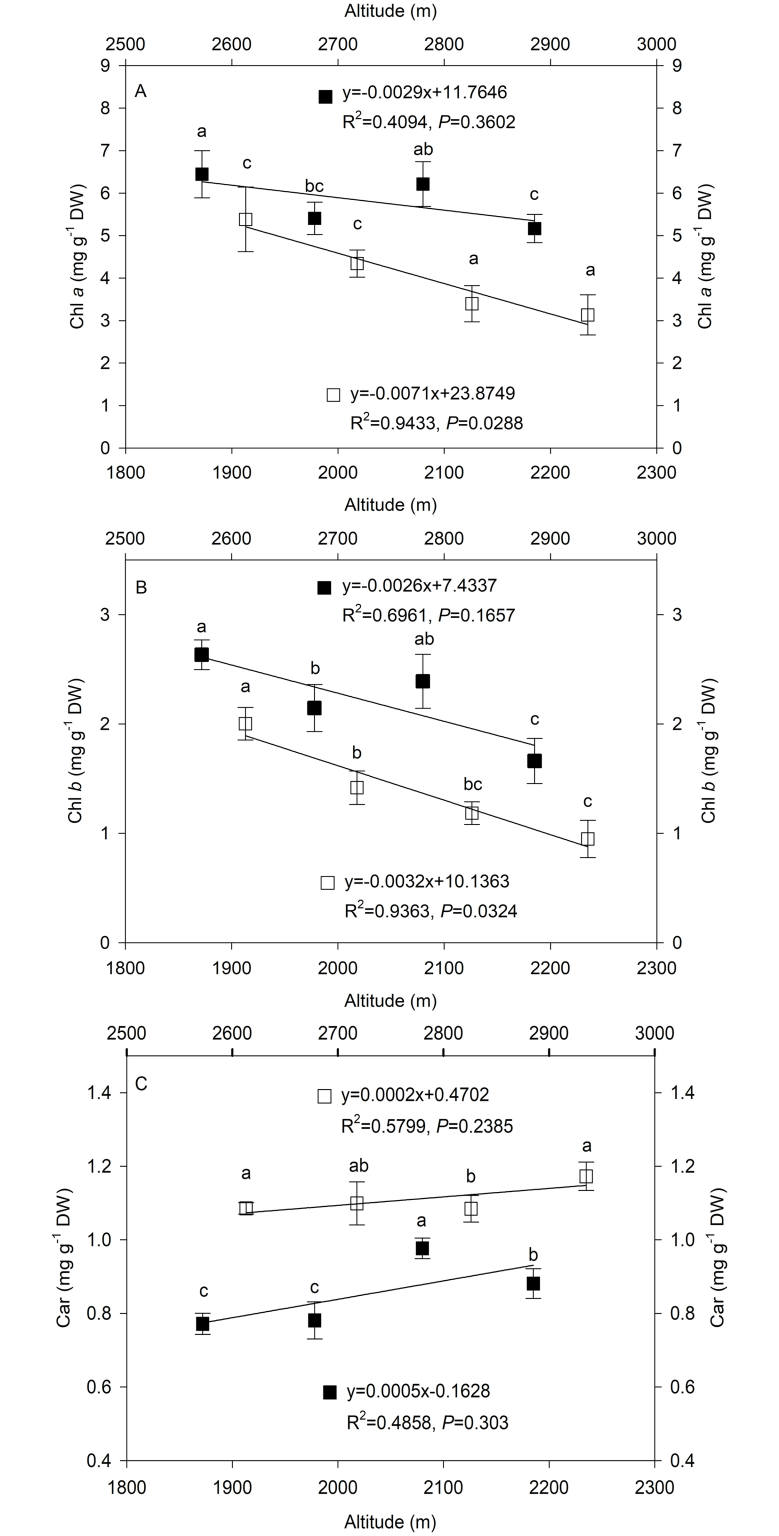
Changes in the chlorophyll *a* (A), chlorophyll *b* (B) and total carotenoid (C) contents along altitudinal gradients. The symbols represent Minhe County (MH = ■) and Huangzhong County (HZ = □). The trendline, regression formula, coefficient of determination and P-value for sites in MH and HZ are shown. Values are shown as the mean ± SD of three years. Different letters indicate that the mean values are significantly different among the treatments (α = 0.05).

The effect of altitudinal gradients resulted in a decrease in chlorophyll a (Chl a) and chlorophyll b (Chl *b*) contents and an increase in total carotenoid (Car) contents of *L*. *secalinus* in both MH and HZ (Fig 2). The Chl *a* and Chl *b* contents were higher in MH than in HZ (Fig 2A and 2B). The Chl *a* contents in HZ showed a significant negative correlation with elevation, and the correlation coefficient was -0.971 (Fig 2A). The Chl *b* contents also showed a significant negative correlation with elevation in HZ, and the correlation coefficient was -0.968 (Fig 2B). However, the Car content of *L*. *secalinus* in both MH and HZ increased with altitude (Fig 2C). Overall, the reduction in Chl *a* and Chl *b* contents in high-altitude regions (0.70 and 0.33 mg g^-1^ DW every 100 meters above sea level, respectively) was greater than that in low-altitude regions (0.41 and 0.31 mg g^-1^ DW every 100 meters above sea level, respectively). The increase in Car content in high-altitude regions (0.04 mg g^-1^ DW every 100 meters above sea level) was higher than that in low-altitude regions (0.03 mg g^-1^ DW every 100 meters above sea level).

### Changes in soluble protein content and antioxidant enzyme activities along altitudinal gradients

**Fig 3:**
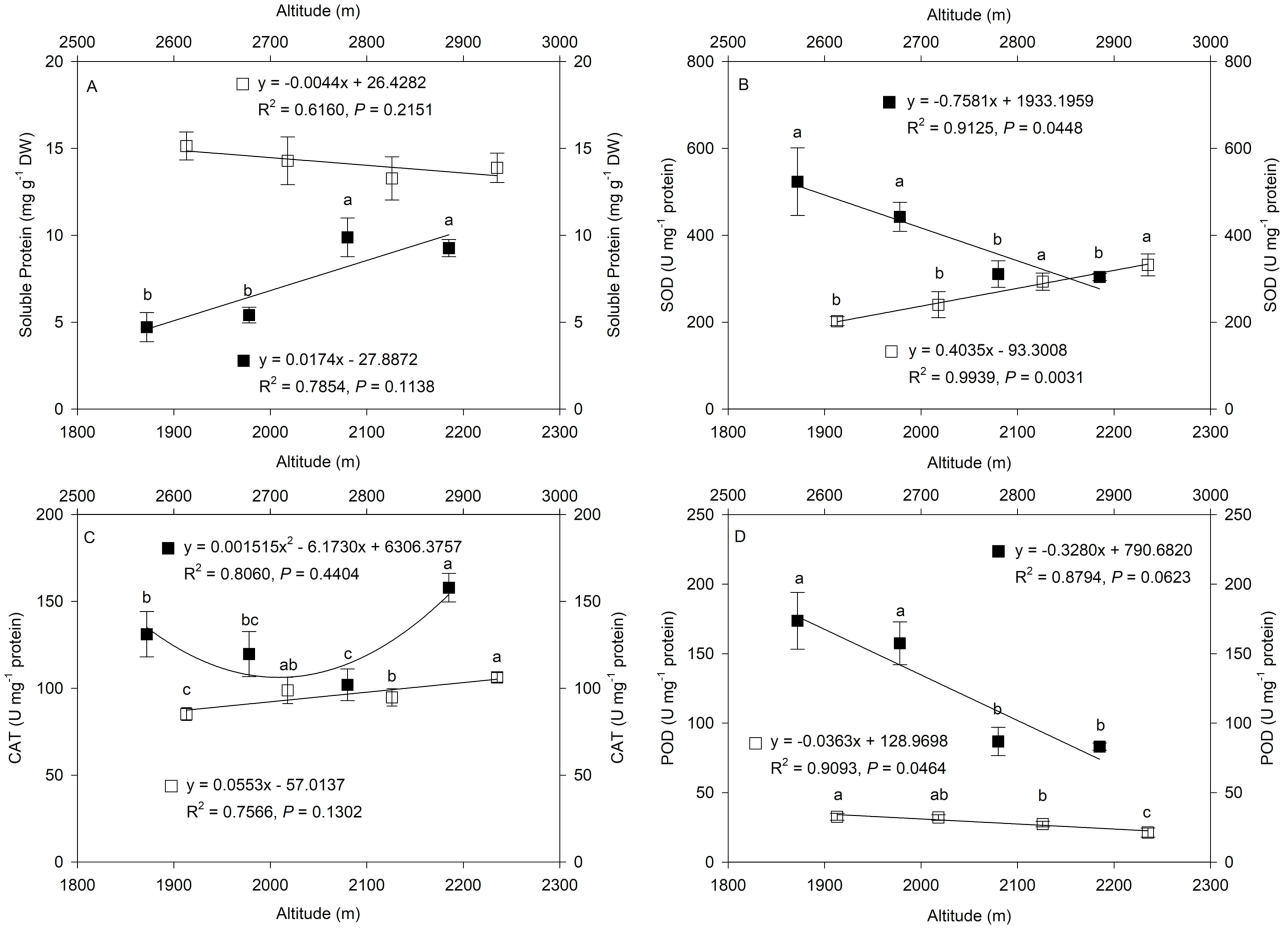
Changes in soluble protein content (A) and superoxide dismutase (SOD) (B), catalase (CAT) (C) and peroxide (POD) (D) activities along altitudinal gradients. The symbols represent Minhe County (MH = ■) and Huangzhong County (HZ = □). The trendline, regression formula, coefficient of determination and P-value for sites in MH and HZ are shown. Values are shown as the mean ± SD of three years. Different letters indicate that the mean values are significantly different among the treatments (α = 0.05).

Higher altitudes affected the soluble protein content and the activities of superoxide dismutase (SOD), catalase (CAT) and peroxidase (POD) in MH and HZ, but these responses varied along an altitudinal gradient (Fig 3). The protein content of *L*. *secalinus* increased significantly with elevation in MH, but no significant differences were detected in HZ; however, the average protein content was noticeably higher in HZ than in MH (Fig 3A). The activities of SOD, CAT and POD were measured to better understand how *L*. *secalinus* copes with oxidative stress along altitudinal gradients. As shown in Fig 3B, the SOD activities decreased significantly with elevation in MH, and the opposite trend was observed in HZ; the correlation coefficients were -0.9552 and 0.9978, respectively. The CAT activities in MH decreased at altitudes of 1187–2080 m and then increased significantly with increasing altitude, and a significant increase was also observed in HZ (Fig 3C). In contrast to the changes in SOD and CAT activities, the POD activity significantly decreased with an increase in altitude at all the sites in both MH and HZ (Fig 3D). In addition, the average SOD, CAT and POD activities in MH were higher than those in HZ.

### Changes in proline and soluble sugar content along altitudinal gradients

**Fig 4:**
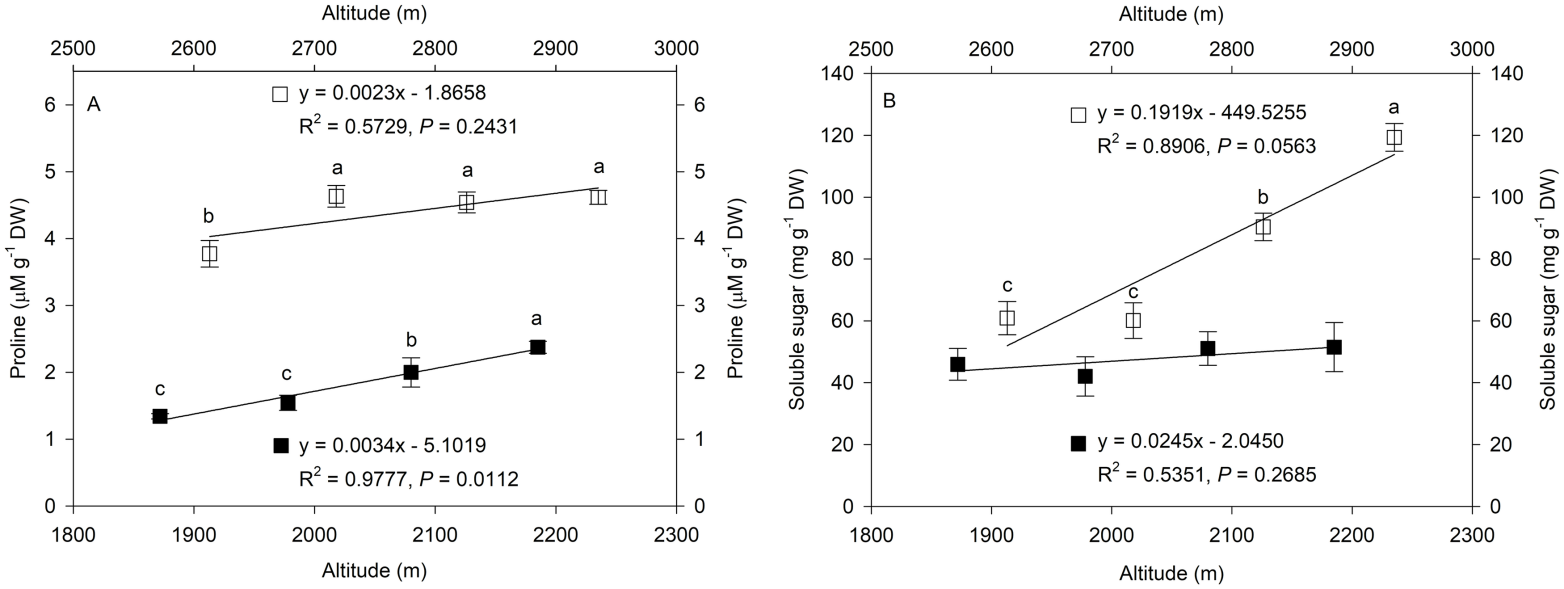
Changes in proline (A) and soluble sugar (B) contents along altitudinal gradients. The symbols represent Minhe County (MH = ■) and Huangzhong County (HZ = □). The trendline, regression formula, coefficient of determination and P-value for sites in MH and HZ are shown. Values are shown as the mean ± SD of three years. Different letters indicate that the mean values are significantly different among the treatments (α = 0.05).

Proline and soluble sugar are reducing agents and play important roles in controlling osmotic adjustments. The proline and soluble sugar contents of *L*. *secalinus* were affected by altitude and exhibited similar increasing trends along altitudinal gradients (Fig 4). The proline content of *L*. *secalinus* showed a significant increase in MH, but there was no significant change from 2718 meters to 2935 meters in HZ (Fig 4A). Compared to the small increase in proline content in HZ (0.26 μM g^-1^ DW every 100 meters above sea level), the increase in MH (0.33 μM g^-1^ DW every 100 meters above sea level) was 1.3 times higher. A large increase in soluble sugar content was observed in HZ from 2718 meters to 2935 meters (Fig 4B). The highest soluble sugar content was observed in HZ4, with an increase of up to 2.0-fold compared to that in HZ1. The soluble sugar contents in MH did not show as drastic an increase as they did in HZ and showed no difference among the four sites in MH.

## Discussion

Various environmental factors on the Qinghai-Tibetan Plateau, including light intensity, photoperiod, UV intensity, temperature, moisture and oxygen, change along altitudinal gradients [25]. Oxygen pressure, precipitation, light intensity and radiation intensity increase as the altitude rises. Meanwhile, temperature and gravity are reduced. Therefore, the impact of altitude on plant growth is the result of the combined action of various factors [26]. The plants grown on the Qinghai-Tibetan Plateau can change in morphological structure, physiology and metabolism in ways that allow them to complete their life cycles, and these changes often have their own regularity, such as the cell membrane, photosynthetic pigments, antioxidant defense system and osmotic adjustments [27, 28]. The adaptation of *Kobresia pygmaea* to the high-elevation environment of the Qinghai-Tibetan Plateau via an immense accumulation of metabolites and proteins related to stresses and life history events mediated by proteins has been confirmed [29].

Stress-induced damage at the cellular level can induce the accumulation of reactive oxygen species (ROS) and lead to membrane lipid peroxidation [30]. Malondialdehyde (MDA) is a final product of lipid peroxidation and has been used widely as an indicator of membrane injury caused by free radicals under various abiotic stresses [31]. Our study found that the MDA contents in leaves of *L*. *secalinus* first increased and then declined along altitudinal gradients in Minhe County (MH); however, these contents showed a significant linear decrease in Huangzhong County (HZ). The decrease in MDA contents may reflect an enhanced ROS scavenging capacity and an alleviation of plasma membrane damage in the high-altitude region, suggesting that *L*. *secalinus* growth on the Qinghai-Tibetan Plateau may have high adaptability to abiotic stresses.

Photosynthetic pigments, which are involved in the absorption and transformation of light, directly affect plant photosynthetic capacity and are sensitive to environmental stresses. Water stress, high light and high temperature can reduce chlorophyll content by slowing chlorophyll synthesis, accelerating decomposition, or damaging chloroplast structures [32, 33]. In the present study, the chlorophyll *a* (Chl *a*) and chlorophyll *b* (Chl *b*) contents decreased with increasing elevation, and this may be an adaptive response of *L*. *secalinus* to avoid oxidative damage by reducing light absorption and thereby preventing the production of ROS. In addition, the Chl *b* content decreased more rapidly than that of Chl *a* in MH and HZ, which was similar to the results observed in evergreen woody species [34].

Carotenoids are a multifunctional complex not only involved in photosynthesis and the composition of light-harvesting complexes (LHCs) but also capable of effectively removing the accumulated ROS to avoid the damage to chloroplasts caused by photooxidation [35, 36]. Carotenoid (Car) contents are known to decrease at high altitudes, but the opposite case has also been observed [26]. In this study, the Car contents increased with elevation, and the magnitude of the increase in high-altitude regions was higher than that in low-altitude regions, suggesting that environmental factors at a higher altitude may promoted the biosynthesis of Car, which is similar to the patterns detected in the skin tissue of peach grown in low- and high-altitude areas [37]. Light is necessary for Car biosynthesis, and the increase in light intensity that accompanies an increase in elevation is also conducive to Car synthesis [38]. Hence, the enhancement in Car contents with increasing altitude may be attributed to the protective function of the pigments in the dissipation of excess energy and scavenging of free radicals [39]. The significant decline in Chl *a* and Chl *b* and the increase in Car in high-altitude regions may be related to the decrease in photosynthesis and increase in photoinhibition [40].

The imbalance of free-radical metabolism under abiotic stresses can trigger an overaccumulation of ROS and cause oxidative damage to plants [41]. To cope with oxidative damage under various adverse conditions, plants have developed an antioxidant defense system that includes antioxidant enzymes such as superoxide dismutase (SOD), catalase (CAT) and peroxidase (POD) [42]. SOD, as a primary scavenger of superoxide anion radicals, can catalyze the conversion of the superoxide anion radical (O_2_·^-^) to H_2_O_2_, and the increase in SOD activity was correlated with increased protection from damage associated with oxidative stress [41, 43]. CAT is among the most effective antioxidant enzymes at scavenging H_2_O_2_ produced by catalyzing the decomposition of H_2_O_2_ into water and oxygen [44], and the enzyme plays a central role in scavenging ROS and maintaining the physiological redox status of organisms [45]. In this study, the decrease in SOD and CAT activities with elevation in MH suggested that the activities of these two enzymes were sufficient to modulate O_2_·^-^and H_2_O_2_ levels [46] and may reflect better adaptability of *L*. *secalinus* along altitudinal gradients. Conversely, the significant increase in SOD and CAT activities with increasing altitude in HZ may be required to cope with oxidative stress by maintaining O_2_·^-^ and H_2_O_2_ at low levels.

Although POD has a similar function to CAT in scavenging H_2_O_2_, its catalyzing reaction requires other substances in plants, such as an electron donor [47]. In this study, the POD activity of *L*. *secalinus* in MH first increased and then decreased from 1978 meters to 2185 meters above sea level, and that in HZ decreased at all sites with an increase in altitude. The initiation of some antioxidant systems may require the reducing activities of some antioxidant enzymes [48]. On the one hand, POD can defend ROS early in senescence; on the other hand, POD can induce the decomposition of chlorophyll and cause lipid peroxidation later in senescence. Thus, the opposing trends of CAT and POD in *L*. *secalinus* may be an adjustment mechanism on the Qinghai-Tibetan Plateau.

Accumulation of proline is an important abiotic stress indicator in higher plants [49]. Proline might involve osmoregulation and act as a cellular osmotic regulator or radical scavenger [43, 50]. In a previous study, there was a strong positive correlation between proline accumulation and plant adaptation [33]. Our results showed a tendency of proline contents of *L*. *secalinus* to increase along altitudinal gradients, which was consistent with the report that free proline content significantly increased in *Potentilla saundersiana* along an altitudinal gradient [51]. The higher concentration of proline accumulated in the leaves of *L*. *secalinus* in HZ compared to that in MH provides a significant protective function and indicates the importance of proline accumulation in plant adaptation.

Soluble protein and soluble sugar are considered to be important osmotic regulators in plants suffering from abiotic stress, which can be induced directly or indirectly by various environmental factors [52]. The accumulation of soluble protein and soluble sugar will increase the concentration of cellular fluid, stabilize cell membranes and reduce osmotic potential [53]. In this study, the protein contents of *L*. *secalinus* increased significantly with elevation in MH, and the average protein content in HZ was noticeably higher than that in MH. In addition, the soluble sugar contents increased significantly from 2718 meters to 2935 meters above sea level, and the average soluble sugar content in HZ was higher than that in MH. The increase in soluble proteins with altitude may raise the functional protein content and ensure cell metabolism. The higher soluble sugar contents in plants of alpine regions may indicate that more carbohydrate or soluble sugar transport is needed to maintain osmotic adjustment. In addition, sucrose can also act as a defense mechanism due to its capacity to react with the hydroxyl radical [54].

## Conclusions

This study provided some supporting evidence for the negative effects of altitudinal gradients on *L*. *secalinus* growth on the Qinghai-Tibetan Plateau, and the adaptation strategies of *L*. *secalinus* to elevation can be attributed to the combined effects of multiple environmental drivers. The reduction in Chl *a* and Chl *b* contents and the increase in Car content can help plants reduce light absorption and avoid photodamage to the chloroplast. The decrease in MDA content associated with lower lipid peroxidation, and the changes of CAT, SOD and POD activities may reflect a higher ROS scavenging capacity in high-altitude regions. The increase in proline and soluble sugar contents with elevation suggests that proline and soluble sugar may play a key role in osmotic adjustment in plants of alpine regions. Further studies are needed to fully understand how altitude affects *L*. *secalinus* growth on the Qinghai-Tibetan Plateau.

## References

[1] Ahmad KS, Hameed M, Hamid A, Nawaz F, Kiani BH, Ahmad MSA, et al Beating cold by being tough: impact of elevation on leaf characteristics in Phleum himalaicum Mez. endemic to Himalaya. Acta Physiologiae Plantarum. 2018;40(3):56 10.1007/s11738-018-2637-4

[2] Barry RG. Mountain Weather and Climate. London Methuen; 1981.

[3] Körner C. The use of ‘altitude’ in ecological research. Trends Ecol Evol. 2007;22(11):569–74. 10.1016/j.tree.2007.09.006 17988759

[4] Shepherd T, Griffiths DW. The effects of stress on plant cuticular waxes. New Phytol. 2006;171(3):469–99. 10.1111/j.1469-8137.2006.01826.x 16866954

[5] Körner C. Alpine plant life: functional plant ecology of high mountain ecosystems Berlin/Heidelberg: Springer Verlag; 2003.

[6] Rana MS, Samant SS, Rawat YS. Plant communities and factors responsible for vegetation pattern in an alpine area of the northwestern Himalaya. Journal of Mountain Science. 2011;8(6):817–26. 10.1007/s11629-011-2078-7

[7] Wu X, Zhang X, Dong S, Cai H, Zhao T, Yang W, et al Local perceptions of rangeland degradation and climate change in the pastoral society of Qinghai-Tibetan Plateau. The Rangeland Journal. 2015;37(1):11–9.

[8] Zhu Pj, Yang L. Ambient UV-B radiation inhibits the growth and physiology of Brassica napus L. on the Qinghai-Tibetan plateau. Field Crops Research. 2015;171:79–85. 10.1016/j.fcr.2014.11.006.

[9] Ma WL, Shi PL, Li WH, He YT, Zhang XZ, Shen ZX, et al Changes in individual plant traits and biomass allocation in alpine meadow with elevation variation on the Qinghai-Tibetan Plateau. Sci China-Life Sci. 2010;53(9):1142–51. 10.1007/s11427-010-4054-9 21104375

[10] Huang B, DaCosta M, Jiang Y. Research Advances in Mechanisms of Turfgrass Tolerance to Abiotic Stresses: From Physiology to Molecular Biology. Critical Reviews in Plant Sciences. 2014;33(2–3):141–89. 10.1080/07352689.2014.870411

[11] Wright IJ, Reich PB, Cornelissen JHC, Falster DS, Groom PK, Hikosaka K, et al Modulation of leaf economic traits and trait relationships by climate. Global Ecology and Biogeography. 2005;14(5):411–21. 10.1111/j.1466-822x.2005.00172.x

[12] Xuehua YE, Feihai YU, Dong M. A Trade-off Between Guerrilla and Phalanx Growth Forms in Leymus secalinus Under Different Nutrient Supplies. Ann Bot. 2006;98(1):187 10.1093/aob/mcl086 16687430 PMC2803537

[13] Kang J, Zhao W, Zhao M. Remediation of blowouts by clonal plants in Maqu degraded alpine grasslands of northwest China. J Plant Res. 2017;130(2):291–9. 10.1007/s10265-016-0884-2 . 27909827

[14] Zhu Y, Dong M, Huang Z. Caryopsis germination and seedling emergence in an inland dune dominant grass Leymus secalinus. Flora—Morphology, Distribution, Functional Ecology of Plants. 2007;202(3):249–57.

[15] He C, Zhang Q, Li Y, Li X, Shi P. Zoning grassland protection area using remote sensing and cellular automata modeling—A case study in Xilingol steppe grassland in northern China. J Arid Environ. 2005;63(4):814–26.

[16] Heath RL, Packer L. Effect of light on lipid peroxidation in chloroplasts. Biochem Biophys Res Commun. 1965;19(6):716–20. 5840698 10.1016/0006-291x(65)90316-5

[17] Wellburn AR, Lichtenthaler H. Formulae and Program to Determine Total Carotenoids and Chlorophylls A and B of Leaf Extracts in Different Solvents: Springer Netherlands; 1984 9–12 p.

[18] Bradford M. A rapid and sensitive method for the quantitation of microgram quantities of protein utilizing the principle of protein-dye binding. Anal Biochem. 1976;72(1–2):248–54. 942051 10.1016/0003-2697(76)90527-3

[19] Giannopolitis CN, Ries SK. Superoxide dismutases: I. Occurrence in higher plants. Plant physiology. 1977;59(2):309–14. 16659839 10.1104/pp.59.2.309 PMC542387

[20] Maehly AC. The assay of catalases and peroxidases Methods Biochem Anal: John Wiley & Sons, Inc.; 2006 p. 357–424. 10.1002/9780470110171.ch14 13193536

[21] Raza S, Athar H, Ashraf M, Hameed A. Glycinebetaine-induced modulation of antioxidant enzymes activities and ion accumulation in two wheat cultivars differing in salt tolerance. Environ Exp Bot. 2007;60(3):368–76.

[22] Zaharieva T, Yamashita K, Matsumoto H. Iron Deficiency Induced Changes in Ascorbate Content and Enzyme Activities Related to Ascorbate Metabolism in Cucumber Roots. Plant Cell Physiol. 1999;40(3):273–80.

[23] Bates LS, Waldren RP, Teare ID. Rapid determination of free proline for water-stress studies. Plant Soil. 1973;39(1):205–7.

[24] Dreywood R. Qualitative test for carbohydrate material. Industrial & Engineering Chemistry Analytical Edition. 1946;18(8):499-.

[25] Chen BX, Zhang XZ, Tao J, Wu JS, Wang JS, Shi PL, et al The impact of climate change and anthropogenic activities on alpine grassland over the Qinghai-Tibet Plateau. Agr Forest Meteorol. 2014;189(189):11–8.

[26] Ahmad KS, Hameed M, Fatima S, Ashraf M, Ahmad F, Naseer M, et al Morpho-anatomical and physiological adaptations to high altitude in some Aveneae grasses from Neelum Valley, Western Himalayan Kashmir. Acta Physiologiae Plantarum. 2016;38(4). 10.1007/s11738-016-2114-x

[27] Cui GX, Wei XH, Degen AA, Wei XX, Zhou JW, Ding LM, et al Trolox-equivalent antioxidant capacity and composition of five alpine plant species growing at different elevations on the Qinghai-Tibetan Plateau. Plant Ecology & Diversity. 2016;9(4):387–96.

[28] Qin JH, Liu Q. Impact of seasonally frozen soil on germinability and antioxidant enzyme activity of Picea asperata seeds. Can J For Res. 2009;39(4):723–30.

[29] Li X, Yang YQ, Ma L, Sun XD, Yang SH, Kong XX, et al Comparative Proteomics Analyses of Kobresia pygmaea Adaptation to Environment along an Elevational Gradient on the Central Tibetan Plateau. PloS one. 2014;9(6):13 10.1371/journal.pone.0098410 24887403 PMC4041879

[30] Demiral T, Turkan I. Comparative lipid peroxidation, antioxidant defense systems and proline content in roots of two rice cultivars differing in salt tolerance. Environ Exp Bot. 2005;53(3):247–57.

[31] Alexieva V, Sergiev I, Mapelli S, Karanov E. The effect of drought and ultraviolet radiation on growth and stress markers in pea and wheat. Plant, Cell & Environment. 2001;24(12):1337–44.

[32] Hazrati S, Tahmasebi-Sarvestani Z, Modarres-Sanavy SAM, Mokhtassi-Bidgoli A, Nicola S. Effects of water stress and light intensity on chlorophyll fluorescence parameters and pigments of Aloe vera L. Plant Physiology and Biochemistry. 2016;106:141–8. 10.1016/j.plaphy.2016.04.046 27161580

[33] Ashraf M, Harris PJC. Photosynthesis under stressful environments: An overview. Photosynthetica. 2013;51(2):163–90. 10.1007/s11099-013-0021-6

[34] Li Y, Yang DM, Xiang S, Li GY. Different responses in leaf pigments and leaf mass per area to altitude between evergreen and deciduous woody species. Australian Journal of Botany. 2013;61(6):424–35. 10.1071/bt13022

[35] Armstrong GA, Hearst JE. Carotenoids 2: Genetics and molecular biology of carotenoid pigment biosynthesis. FASEB J. 1996;10(2):228–37. . 8641556 10.1096/fasebj.10.2.8641556

[36] Demmig-Adams B, Iii WWA. Carotenoid composition in sun and shade leaves of plants with different life forms. Plant Cell & Environment. 1992;15(4):411–9.

[37] Karagiannis E, Tanou G, Samiotaki M, Michailidis M, Diamantidis G, Minas IS, et al Comparative Physiological and Proteomic Analysis Reveal Distinct Regulation of Peach Skin Quality Traits by Altitude. Frontiers in plant science. 2016;7:1689 10.3389/fpls.2016.01689 . 27891143 PMC5102882

[38] Simkin AJ, Zhu C, Kuntz M, Sandmann G. Light-dark regulation of carotenoid biosynthesis in pepper (Capsicum annuum) leaves. J Plant Physiol. 2003;160(5):439–43. 10.1078/0176-1617-00871 12806770

[39] H M, N KK. The violaxanthin cycle protects plants from photooxidative damage by more than one mechanism. Proc Natl Acad Sci U S A. 1999;96(15):8762–7. 10411949 10.1073/pnas.96.15.8762 PMC17590

[40] Demmig-Adams B, Adams W. W. III. Photoprotection and Other Responses of Plants to High Light Stress. Annual Review of Plant Physiology and Plant Molecular Biology. 1992;43(1):599–626.

[41] Asada K. THE WATER-WATER CYCLE IN CHLOROPLASTS: Scavenging of Active Oxygens and Dissipation of Excess Photons. Annu Rev Plant Physiol Plant Mol Biol. 1999;50(1):601–39. 10.1146/annurev.arplant.50.1.601 . 15012221

[42] Foyer CH, Noctor G. Oxidant and antioxidant signalling in plants: a re-evaluation of the concept of oxidative stress in a physiological context. Plant Cell Environ. 2005;28(8):1056–71. 10.1111/j.1365-3040.2005.01327.x

[43] Smirnoff N. The role of active oxygen in the response of plants to water deficit and desiccation. New Phytol. 2006;125(1):27–58. 10.1111/j.1469-8137.1993.tb03863.x 33874604

[44] Scandalios J. Oxygen stress and superoxide dismutases. Plant Physiol. 1993;101(1):7–12. 12231660 10.1104/pp.101.1.7 PMC158641

[45] Cho UH, Seo NH. Oxidative stress in Arabidopsis thaliana exposed to cadmium is due to hydrogen peroxide accumulation. Plant Sci. 2005;168(1):113–20.

[46] Wang Y, He W, Huang H, An L, Wang D, Zhang F. Antioxidative responses to different altitudes in leaves of alpine plant Polygonum viviparum in summer. Acta Physiologiae Plantarum. 2009;31(4):839–48. 10.1007/s11738-009-0300-9

[47] Abdel Latef AA, Tran LS. Impacts of Priming with Silicon on the Growth and Tolerance of Maize Plants to Alkaline Stress. Front Plant Sci. 2016;7:243 10.3389/fpls.2016.00243 27014283 PMC4785188

[48] Foyer CH, Halliwell B. The presence of glutathione and glutathione reductase in chloroplasts: A proposed role in ascorbic acid metabolism. Planta. 1976;133(1):21 10.1007/BF00386001 24425174

[49] Paleg L, Aspinall D. The physiology and biochemistry of drought resistance in plants: Academic Press; 1981.

[50] Manivannan P, Jaleel C, Sankar B, Kishorekumar A, Somasundaram R, Lakshmanan G, et al Growth, biochemical modifications and proline metabolism in Helianthus annuus L. as induced by drought stress. Colloids Surf B Biointerfaces. 2007;59(2):141–9. 10.1016/j.colsurfb.2007.05.002 17560769

[51] Ma L, Sun X, Kong X, Galvan JV, Li X, Yang S, et al Physiological, biochemical and proteomics analysis reveals the adaptation strategies of the alpine plant Potentilla saundersiana at altitude gradient of the Northwestern Tibetan Plateau. Journal of proteomics. 2015;112:63–82. Epub 2014/09/03. 10.1016/j.jprot.2014.08.009 . 25181701

[52] Bartels D, Sunkar R. Drought and salt tolerance in plants. Crit Rev Plant Sci. 2005;24(1):23–58.

[53] Basu PS, Berger JD, Turner NC, Chaturvedi SK, Ali M, Siddique KHM. Osmotic adjustment of chickpea (Cicer arietinum) is not associated with changes in carbohydrate composition or leaf gas exchange under drought. Annals of Applied Biology. 2007;150(2):217–25.

[54] Van d E W, Valluru R. Sucrose, sucrosyl oligosaccharides, and oxidative stress: scavenging and salvaging? Journal of Experimental Botany. 2009;60(1):9–18. 10.1093/jxb/ern297 19036839

